# SATB1 Mediates Long-Range Chromatin Interactions: A Dual Regulator of Anti-Apoptotic *BCL2* and Pro-Apoptotic *NOXA* Genes

**DOI:** 10.1371/journal.pone.0139170

**Published:** 2015-09-30

**Authors:** Yin Yang, Zongdan Wang, Luan Sun, Lipei Shao, Nan Yang, Dawei Yu, Xin Zhang, Xiao Han, Yujie Sun

**Affiliations:** 1 Key Laboratory of Human Functional Genomics of Jiangsu Province, Nanjing medical University, Nanjing, PR China; 2 Department of Cell Biology, Nanjing Medical University, Nanjing, PR China; 3 Department of Physiology and Stephenson Cancer Center, University of Oklahoma Health Sciences Center, University of Oklahoma, Norman, Oklahoma, United States of America; 4 Collaborative Innovation Center for Cancer Personalized Medicine, Jiangsu Key Lab of Cancer Biomarkers, Prevention & Treatment, Cancer Center, Nanjing Medical University, Nanjing, PR China; Emory University, UNITED STATES

## Abstract

Aberrant expression of special AT-rich binding protein 1 (SATB1), a global genomic organizer, has been associated with various cancers, which raises the question of how higher-order chromatin structure contributes to carcinogenesis. Disruption of apoptosis is one of the hallmarks of cancer. We previously demonstrated that SATB1 mediated specific long-range chromosomal interactions between the mbr enhancer located within 3’-UTR of the *BCL2* gene and the promoter to regulate BCL2 expression during early apoptosis. In the present study, we used chromosome conformation capture (3C) assays and molecular analyses to further investigate the function of the SATB1-mediated higher-order chromatin structure in co-regulation of the anti-apoptotic *BCL2* gene and the pro-apoptotic *NOXA* gene located 3.4Mb downstream on Chromosome 18. We demonstrated that the mbr enhancer spatially juxtaposed the promoters of *BCL2* and *NOXA* genes through SATB1-mediated chromatin-loop in Jurkat cells. Decreased SATB1 levels switched the mbr-*BCL2* loop to mbr-NOXA loop, and thus changed expression of these two genes. The SATB1-mediated dynamic switch of the chromatin loop structures was essential for the cooperative expression of the *BCL2* and *NOXA* genes in apoptosis. Notably, the role of SATB1 was specific, since inhibition of SATB1 degradation by caspase-6 inhibitor or caspase-6-resistant SATB1 mutant reversed expression of BCL-2 and NOXA in response to apoptotic stimulation. This study reveals the critical role of SATB1-organized higher-order chromatin structure in regulating the dynamic equilibrium of apoptosis-controlling genes with antagonistic functions and suggests that aberrant SATB1 expression might contribute to cancer development by disrupting the co-regulated genes in apoptosis pathways.

## Introduction

Special AT-rich binding protein 1 (SATB1) is a global genomic organizer that integrates higher-order chromatin organization with regulation of gene expression [1]. It can recruit chromatin-remodeling complexes to the anchored sites, and thereby regulates expression of a number of genes over long distance of chromatin [2]. SATB1 is primarily and highly expressed in thymocytes, and is required for thymocyte development [3] and the activation of Th2 cells [4]. In addition, SATB1 participates in X-chromosome inactivation [5], development of the epidermis and epidermal differentiation [6], differentiation of embryonic stem cells [7], and cortical development [8]. In recent years, aberrant expression of SATB1 has been associated with various cancers, including breast [9], hepatocellular [10], prostate and others [11]. Abnormal SATB1 was also positively correlated with prognostic and pathological properties in breast cancer[12], cutaneous malignant melanoma[13], and gastric cancer [14]. Recent studies in cells with stable or transient knockdown or ectopic overexpression of SATB1 have further emphasized the functional relevance of SATB1 to carcinogenesis [15] and multidrug resistance in cancer cells [14,16]. These observations raised interesting questions of whether and how SATB1-organized higher-order chromatin structure contributes to cancer development.

Disruption of apoptosis is a hallmark of cancer that reinforces tumorigenesis and resistance to cytotoxic cancer therapies [17]. Members of the Bcl-2 family are critical regulators of apoptosis. The intrinsic pathway of apoptosis is governed by the balance between opposing roles of the Bcl-2 protein family, which consists of both pro-apoptotic proteins and anti-apoptotic proteins. These proteins function as ‘life/death switches’ to determine whether the apoptosis pathway should be activated, by integrating diverse inter- and intracellular cues. The Bcl-2 anti-apoptotic proteins maintain equilibrium by holding the pro-apoptotic proteins in check, while induction of the BH3-only pro-survival proteins results in inactivation of the anti-apoptotic proteins, triggering the pro-apoptotic proteins Bax and Bak [18]. Thus, the cell’s fate to undergo apoptosis is determined by interactions between anti-apoptotic proteins and pro-apoptotic proteins. High expression of anti-apoptotic proteins or low expression of pro-apoptotic proteins has been observed in many types of cancer cells [19–21]. These findings highlight the critical role of cooperatively regulating expression of both groups of proteins to maintain the dynamic balance in cell fate decision. Although how these two groups of proteins interact in response to apoptotic stimulation has been extensively studied, little is known about the mechanism of how expression of anti-apoptotic and pro-apoptotic proteins was cooperatively regulated within cells.

Proto-oncogene *BCL2* encodes BCL2 anti-apoptotic protein, a key regulator of apoptosis [22]. Our previous study has found that the major breakpoint region (mbr) located within 3’-UTR of the *BCL2* gene is an enhancer element that contains an SATB1 binding sequence [23,24]. SATB1 is able to regulate BCL2 transcription by mediating specific long-range chromosomal interaction between the mbr enhancer and *BCL2* promoter during the early apoptotic response of Jurkat cells [25]. This finding established a functional link between SATB1-organized higher-order chromatin structure and regulation of the apoptosis pathway for the first time. Our further analyses with 3C assays revealed that the promoter of the *NOXA* gene that is 3.4Mb downstream of the *BCL2* gene on Chromosome 18 also physically interacted with the mbr enhancer, suggesting that the *NOXA* gene is targeted by the mbr enhancer. Consistently, bioinformatic analysis revealed that the *NOXA* gene promoter contained SATB1 binding sequences, indicating a potential functional role of SATB1 in the regulation of NOXA expression.

The *NOXA* gene encodes NOXA, a BH3-only pro-apoptotic protein that functions as sensitizer to neutralize anti-apoptotic BCL2 family proteins [26]. Our primary hypothesis was that SATB1 might cooperatively regulate expression of the anti-apoptotic *BCL2* and pro-apoptotic *NOXA* genes to participate in the control of apoptosis. Aberrant SATB1 expression could disrupt apoptosis by disturbing the balance between anti-apoptotic and pro-apoptotic genes, and thus contribute to cancer development. To test this hypothesis, we focused on two questions: 1) Whether SATB1-mediated long-range chromatin conformation is involved in the regulation of cooperative expression of the BCL2 and NOXA genes, and 2) whether altered SATB1 expression changes the cellular apoptotic response by switching the balance between BCL2 and NOXA.

## Materials and Methods

### Cell culture and induction of apoptosis

Human T lymphoid cell line Jurkat which is kindly supplied by Dr. Krontiris’ Laboratory at City of Hope National Medical Center in Los Angeles, USA were maintained in RPMI 1640 medium supplemented with 10% FBS, 10 mM HEPES, 100unit/ml penicillin and 100mg/ml streptomycin and cultured at 37°C in a humidified atmosphere of 5% CO2. Cells were passaged every 2–3 days for exponential growth. For apoptosis assay, Jurkat cells were exposed to 5μM camptothecin and the equal volume of DMSO as the vehicle control. In another assay, Jurkat cells were preincubated with 10μM Z-VEID-fmk for 30 min for prevention the influence of protease then induced Apoptosis.

### Knockdown SATB1 by RNAi in Jurkat cells

SATB1-specific sh-RNA plasmids were prepared as described in our previous article [24]. The SATB1 shRNA sequence was: 5’-GTCCACCTTGTCTTCTCTC-3’. The non-specific shRNA sequence was: 5’-ACGTGACACGTTCGGAGAA-3’. Jurkat cells were transiently transfected with SATB1 interference plasmids or control plasmids using electroporator. The extent of shRNA-mediated inhibition of SATB1 and its effect on BCL2 and NOXA expression were evaluated by Western blot analysis and RT-RCR, respectively.

### Western blot analysis

Cellular protein was isolated using lysis buffer (50 mM Tris, pH7.4, 0.5% NP-40 and 0.01% SDS) containing protease inhibitors. After boiling for 5min in loading buffer, equal amounts (30μg/lane) of proteins were fractionated on SDS-PAGE gels and transferred to polyvinylidene difluoride membranes. Non-specific protein interactions were blocked by incubation in 5% nonfat dry milk in TST buffer (50 mM Tris-HCl, 150mM NaCl, 0.05% Tween 20, and pH7.6) at room temperature for 1h. Membranes were then incubated with anti-SATB1 and anti-β-Actin antibody (Sigma) in fresh blocking buffer at 4°C overnight. After washing with PBS containing 0.1% (v/v) Tween20, the membranes were incubated with peroxidase-conjugated individual secondary antibodies for 1h at room temperature. The blots were developed with ECL reagent (Amersham Biosciences). Prestained markers (NEB) were used as internal molecular weight standards.

### RNA isolation and real-time RCR

Total RNA was isolated with Trizol reagent (Invitrogen) according to the manufacturer’s protocol. cDNA was synthesized from total RNA (1μg) using applied biosystem cDNA Synthesis Kit according to the Manufacturer’s instructions. The reaction was incubated at 25°C for 10 min, then applied to 37°C for 2 hour, and finally 85°C for 5min. The reactions were stored at -20°C before use. The real-time PCR amplification conditions were 95°C for 10 min, followed by 40 cycles of denaturation at 95°C for 15 s, annealing at 60°C for 1 min. Primers are listed in supporting information: S1 Table

### Generation of caspase-6 resistant mutant SATB1 expression vector

To construct the plasmid SATB1-D254A (aspartate mutant to alanine at position 254 in the human SATB1 primary sequence) which is resistant to caspase-6 cleavage, the SATB1 overexpression plasmid pEGFP-C1-SATB1 (generous gifts from Dr. Krontiris laboratory) was used by using the QuikChange® Site-Directed Mutagenesis Kit (Stratagene). The primer is as follows with the mutated bases in underline: 5’-GATATGATGGTTGAAATGGCTAGTCTTTCTGAGCTATC-3’. Plasmid was confirmed by sequencing (BGI).

### Assay for apoptosis

Apoptosis assays were performed using two Apoptosis Detection Kits (Keygentech) according to the manufacturer’s instructions. Briefly, cells were collected and washed twice with PBS, gently resuspended in binding buffer. Annexin V-FITC and propidium iodide (PI) were then added and incubated in the dark for 15min at room temperature, followed by immediate analysis on FACScan (Becton Dickinson). Another apoptosis detection kit is Annexin V-PE/7-AAD, and the manufacture instruction is similar to the protocol above. The percentage of apoptosis was computed using Cell Quest software (Becton Dickinson).

### Chromatin Immunoprecipitation assay and Quantification of ChIP assay

ChIP assay was conducted with the ChIP assay kit essentially as described in the manufacturer (Upstate). Detailed experimental procedures are described in our previous report article [25]. For quantitative analysis of ChIP products, real-time PCR was carried out according to ROCHE manufacturer's instructions using SYBR Green real-time PCR Master Mix. Primers designed for specifically amplifying NOXA-SBS1, BCL2-SBS1 and mbr of BCL2 were listed in supporting information: S1 Table. Standard curves for relative quantification were calculated using 1:4, 1:16, 1:64, and 1:256 dilutions of input samples. All PCR signals from immno-precipitation samples were referred to their respective input standard curve to normalize differences in cell number and primer efficiency. Real-time PCR datas were analyzed according to the methodology described in a report. ΔCt values were first calculated using the formula: ΔCt = Ct (sample)–Ct (input), from which ΔΔCt (ΔCt experiment sample–ΔCt negative control) and fold difference (2(–ΔΔCt treatment)/ 2(–ΔΔCt control) were derived.

### Chromosome Conformation Capture (3C)

The 3C protocol was adapted from published article with modifications. Detailed experimental procedures are described in our previous article [25]. Quantitative real-time PCR was performed, in the presence of SYBR Green, with appropriate primers from purified DNA as well as the control template. To accurately determine the DNA concentration of a 3C sample, 20-dilutions of each 3C sample was first analyzed by real-time PCR using ChIP BCL2-SBS1 primers and the DNA concentration was calculated according to the standard curve that was established using reference genomic DNA with known concentration. Samples were then subsequently adjusted to a concentration of 50ng/μl. For each primer pair successive 4-fold (PGK1) or 2-fold dilutions of the random ligation standard were used to make a calibration curve for determining a relative quantity of the corresponding ligation product in a 3C sample. The ligation efficiency between the samples were corrected by the interaction between two *Ase* I fragments or *Hind* III fragments within the ubiquitously expressed PGK1 locus that had been checked to be transcribed stably in our experiment systems.

## Results

### 1. SATB1 bound to the promoters of both *NOXA* and *BCL2* genes and mbr enhancer.

**Fig 1:**
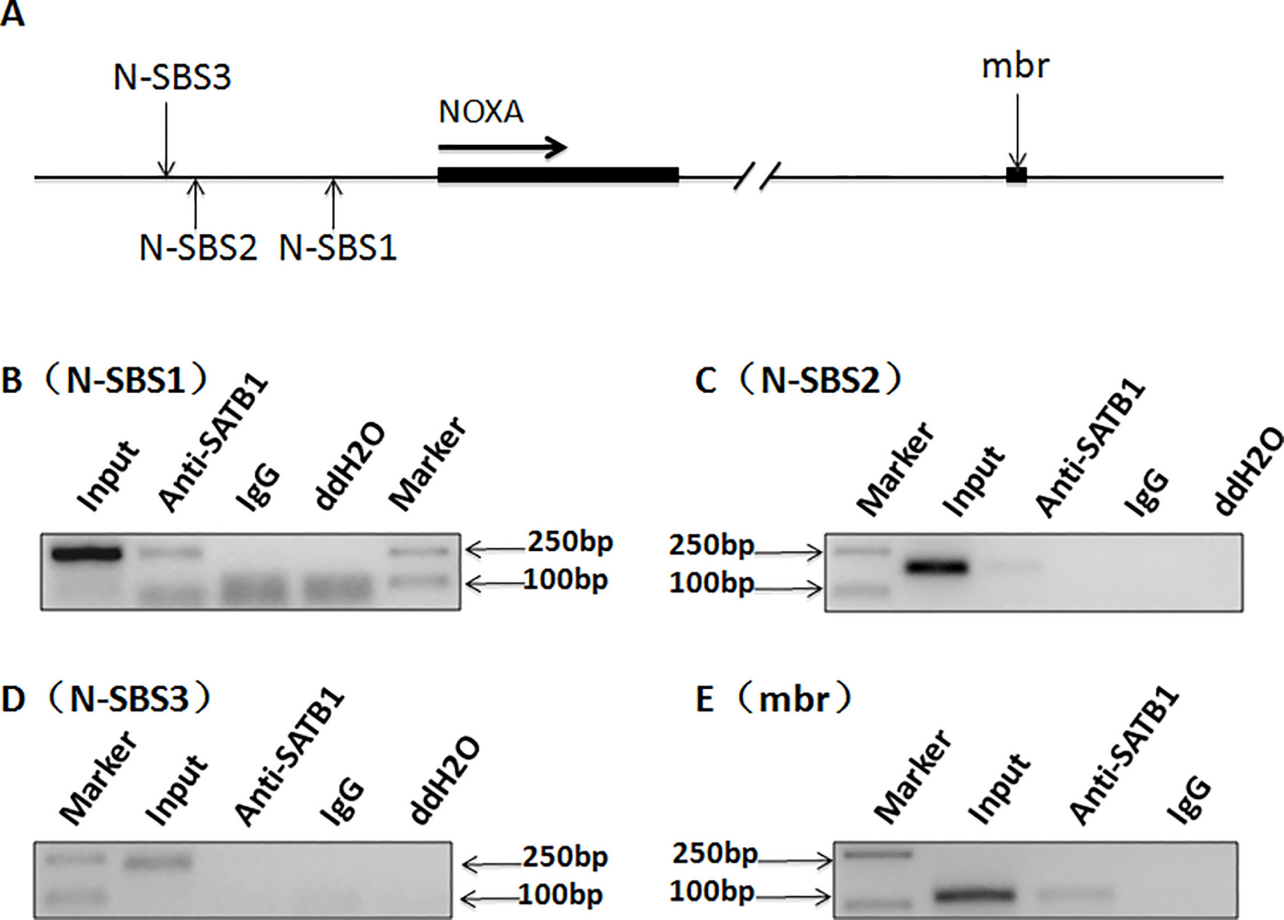
ChIP analysis of SATB1 binding on the NOXA promoter region and the mbr element *in vivo*. Diagram shows the relative positions of three SATB1 binding sites(N-SBS1、N-SBS2、N-SBS3) in NOXA promoter region and mbr(A). The results showed that NOXA-SBS1 sequence located 2.4kb relative to the translational start site were specifically immunoprecipitated with anti-SATB1 (B, indicating that SATB1 binds to this sequence *in vivo*. The SATB1 binding to SBS2 was very weak (C. No SATB1 binding to SBS3 was detected (D. As confirmed earlier, SATB1 bound to the mbr (E). The input represented 1% of total DNA used in immunoprecipitation. Non-specific IgG was used as a negative control. The PCR products were visualized by ethidium bromide staining of a 1.5% agarose gel.

**Fig 2:**
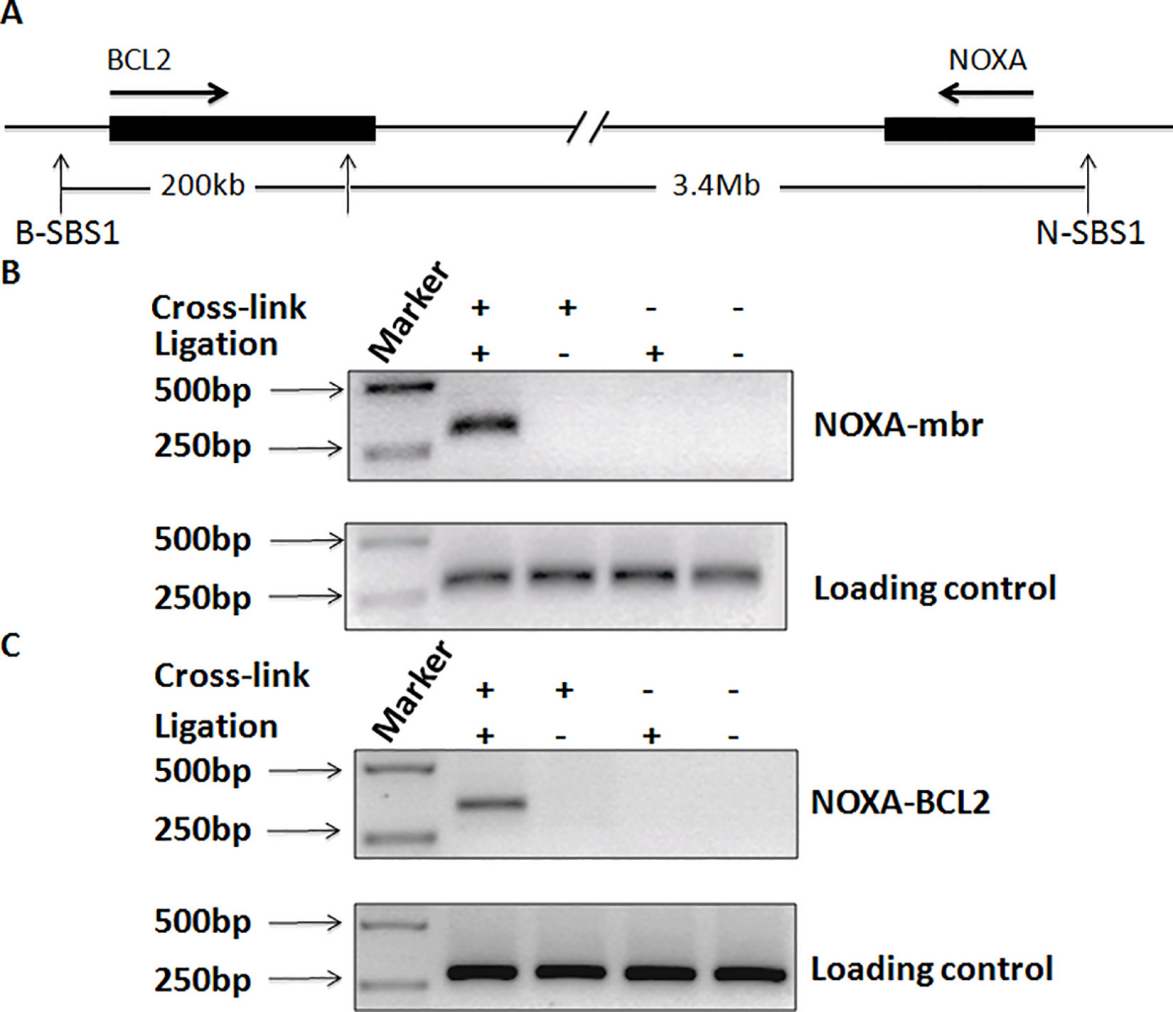
3C assays to detect physical interactions of the mbr-NOXA and NOXA-BCL2. Diagram shows the relative positions of the BCL2 gene, NOXA gene, and mbr element in chromosome 18(A). Physical interactions of the mbr-NOXA (B, upper panel) and NOXA-BCL2 (C, upper panel) genes were identified by PCRs which detected hybrid fragments with specific primers. PCR products from HindIII-digested crosslinked chromatin without ligation and non-crosslinked genomic DNA, with or without ligation, were used as negative controls. We used the PCR products from genomic DNA that was not cut by any restriction enzyme as the loading controls (B & C, lower panels).

We have demonstrated that the SATB1-mediated long-range chromatin interaction between the mbr enhancer and BCL2 promoter is critical to regulating BCL2 expression in the cellular apoptotic response [25]. To investigate whether SATB1-mediated long-range chromatin interaction is involved in co-regulation of anti-apoptotic and pro-apoptotic genes, we used Genomatix Software (Genomatix Software, http://www.genomatix.de/index.html) to analyze the promoter regions of Bcl2 family members for the binding sites of SATB1). The *NOXA* gene located 3.4Mb downstream of the *BCL2* gene was of particular interest. NOXA has 3 sequences that are possible SATB1 binding sites. These sequences are NOXA-SBS1 to NOXA-SBS3, and are located within the 3.5kb region upstream of the NOXA transcription start site. The illustrations of three SATB1 binding sites in NOXA promoter have been shown in Fig 1. We used ChIP assays with the anti-SATB1 antibody to test SATB1 binding on the NOXA promoter. NOXA-SBS1 sequence located 2.4kb relative to the translational start site were found to be specifically immunoprecipitated with anti-SATB1 (Fig 2), indicating that SATB1 binds to this sequence *in vivo*. The precipitated products were weak for SBS2. No specific precipitate was detected for SBS3 (Fig 2). As expected and confirmed in our previous work, SATB1 also bound to the mbr enhancer (Fig 2). Given that SATB1 is a critical factor in regulating long-range interactions between the mbr and BCL2 promoter [25], the binding of SATB1 to the NOXA promoter strongly suggested that SATB1 not only mediated an interaction between the mbr and the BCL2 promoter, but also was involved in the mbr-NOXA chromatin interaction.

### 2. The promoters of both NOXA and BCL2 genes interacted with the mbr enhancer through chromatin loop structures.

Then, we investigated whether NOXA promoter participated in promoter-mbr higher-order chromatin loop structure using a 3C assay, which is able to indicate whether two remote genomic sequences interact in the nucleus. The PCR products derived from the 3C library showed that the *NOXA* gene promoter juxtaposed with the mbr enhancer and *BCL2* gene promoter (Fig 2). The specificity of the PCR products was validated by DNA sequencing (Supporting information: S1 and S2 Figs). These results indicated that the promoter of NOXA physically interacted with the mbr enhancer over 3.4Mb and might form a promoter-promoter-mbr complex with the BCL2 promoter and the mbr enhancer in nucleus through higher-order chromatin structures.

### 3. SATB1 level was critical for the chromatin loop conversion and the cooperative expression of the BCL2 and NOXA genes.

**Fig 3:**
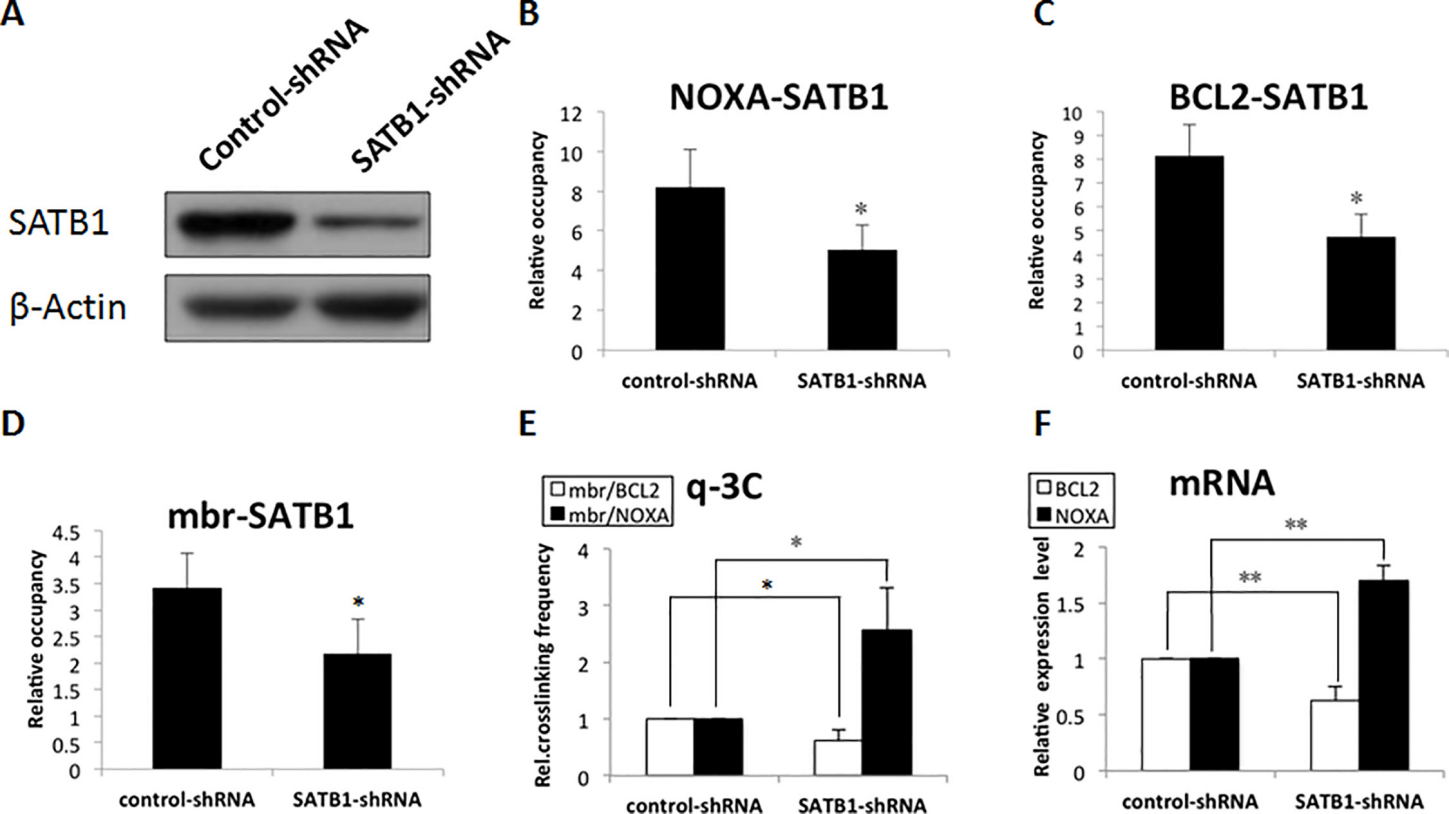
SATB1 was a critical factor for the chromatin loop conversion and the cooperative expression of BCL2 and NOXA genes in Jurkat cells. Jurkat cells were transiently transfected with control-shRNA or SATB1-shRNA plasmids, and targeting efficiency was confirmed by Western blot analysis (A). SATB1 knockdown significantly reduced the association of SATB1 with NOXA promoter (B), BCL2 promoter (C), and mbr (D), as revealed by q-ChIP. Quantitative 3C assays further showed that reduction of SATB1 binding significantly increased mbr-NOXA interactions and dramatically decreased the mbr-BCL2 interactions (E). RT–PCR analysis confirmed the BCL2 mRNA level was decreased while the NOXA mRNA level was increased, keeping pace with the conversion of the mbr-promoter loops; we normalized all the genes by using actin control for quantity. (F). These results indicated that reduced SATB1 switched the mbr-BCL2 chromatin loop to the mbr-NOXA chromatin loop, which was involved in cooperative regulation of the NOXA and BCL2 genes.

To clarify whether SATB1 was involved in the cooperative regulation of NOXA and BCL2 expression by changing higher-order chromatin structure, we examined the correlation between the SATB1 binding on the gene promoters and mbr enhancer and the frequency of mbr-NOXA and mbr-BCL2 chromatin loops. The expression of SATB1 was knocked down in Jurkat cells with plasmids expressing short hairpin RNAs (shRNA). Targeting efficiency was confirmed by Western blot analysis (Fig 3). The effects of SATB1 knockdown were determined by quantitative ChIP assay (q-ChIP). As indicated in Fig 3, SATB1 knockdown significantly reduced the occupation of SATB1 on NOXA promoter. The knockdown-induced reduction of SATB1 binding on the BCL2 promoter region and the mbr enhancer was confirmed (Fig 3). Notably, the reduced SATB1 binding significantly increased mbr-NOXA interactions, while dramatically decreased the mbr-BCL2 interactions (Fig 3), indicating that SATB1 was involved in the dynamic conversion between the mbr-BCL2 chromatin loop and the mbr-NOXA chromatin loop. Under the same experimental conditions, the transcriptional activities of the BCL2 and NOXA genes were determined by real-time PCR. Our data showed that the change in BCL2 and NOXA mRNA levels kept pace with the conversion of the mbr-promoter loops (Fig 3), indicating that SATB1-mediated mbr-promoter interactions were required for BCL2 and NOXA transcriptional activity. These results suggested that SATB1 was an important determinant for conversion between the mbr-BCL2 chromatin loop and the NOXA chromatin loop, and was involved in cooperative regulation of BCL2 and NOXA gene expression.

### 4. SATB1 regulated the cooperative response of the BCL2 and NOXA genes to apoptosis stimulation by switching the mbr-BCL2 chromatin loop to the mbr-NOXA chromatin loop.

**Fig 4:**
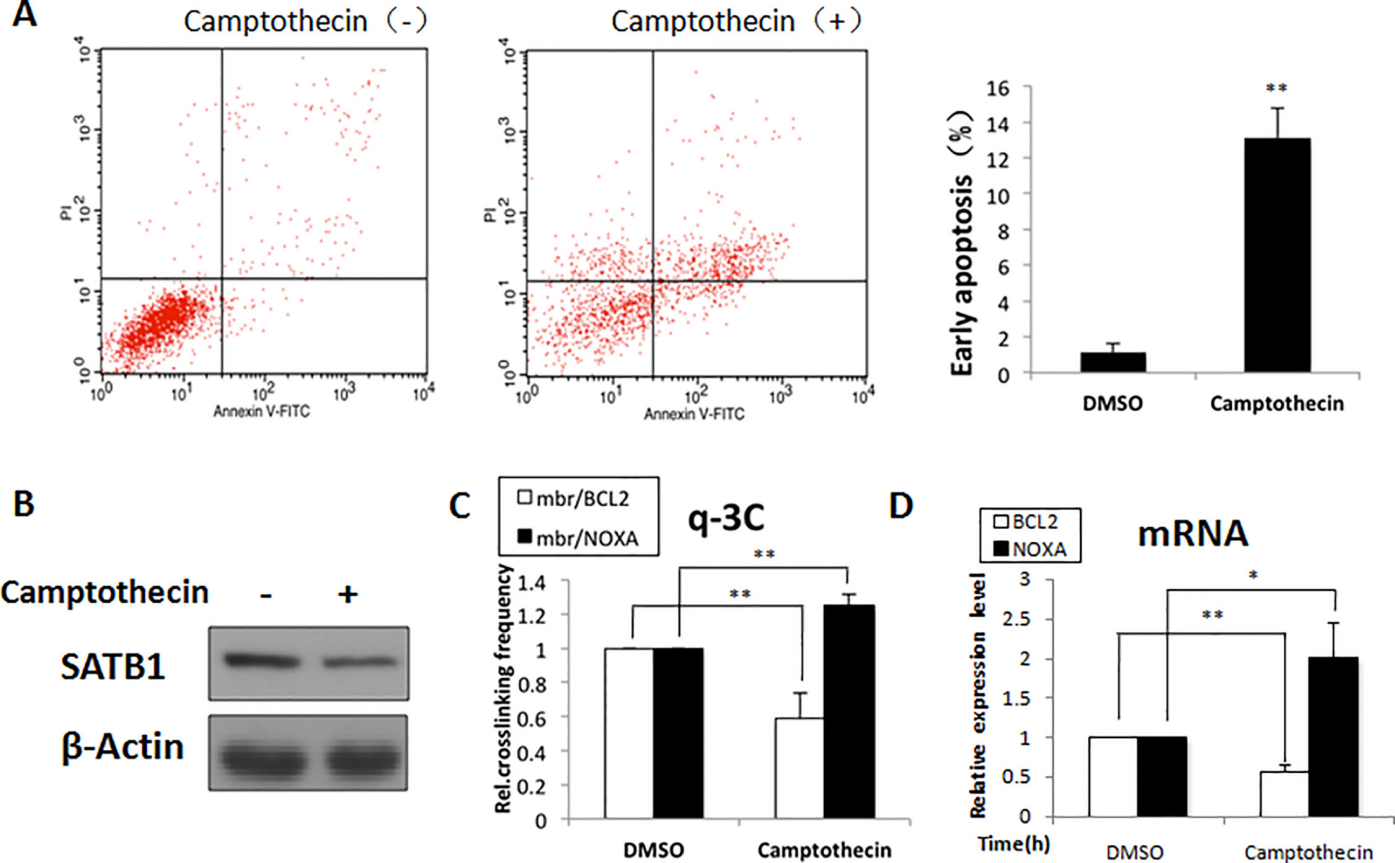
SATB1-induced chromatin loop conversion in the co-regulation of the BCL2 and NOXA genes in cellular response to apoptosis. Jurkat cells were treated with 5μM camptothecin for 2 h to induce apoptosis, and were subsequently harvested. The vehicle control for camptothecin is an equal volume of DMSO. The apoptosis rates were determined by FCM (A) and the degradation of SATB1 was confirmed by Western blot analysis (B). The distal chromatin interactions and gene transcriptional activities were determined by q-3C assays and real-time PCR. The frequency of mbr-BCL2 chromatin loop was significantly reduced, while the frequency of mbr-NOXA chromatin loop was increased in camptothecin-treated cells (C). These findings were coupled with opposite changes in mRNA levels of the BCL2 and NOXA genes (D). These results, together with those described above, suggested that SATB1 was able to co-regulate expression of the BCL2 and NOXA genes at the high-order chromatin structure level.

Apoptosis is a biological process tightly regulated by the pro-apoptotic and anti-apoptotic proteins. To address whether the SATB1-mediated chromatin loop conversion was involved in regulation of the cooperative response of the *BCL2* and *NOXA* genes to apoptosis stimulation, we analyzed the change in chromatin loops, transcriptional activity of the genes, and SATB1 level in Jurkat cells treated with camptothecin. Cells were incubated with 5μM camptothecin and harvested after 2 h, when early apoptosis occurred and SATB1 was degraded to 70%. (Fig 4). The chromatin loop conversion was determined by quantitative 3C assays (q-3C). As indicated in Fig 4, the frequency of interaction between the BCL2 promoter and mbr (mbr-BCL2 chromatin loop) was significantly reduced, while the frequency of interaction between the NOXA promoter and mbr (mbr-NOXA chromatin loop) was increased in camptothecin-treated cells. Consistently, real-time PCR results showed that camptothecin treatment induced opposite changes in mRNA levels of the BCL2 and NOXA genes. The BCL2 mRNA decreased by 50%, while the NOXA mRNA increased by approximately two folds (Fig 4). These data indicated that the.SATB1-mediated conversion between the mbr-BCL2 chromatin loop and the mbr-NOXA chromatin loop was directly involved in the coordinated regulation of the BCL2 and NOXA genes in the cellular apoptotic response.

**Fig 5:**
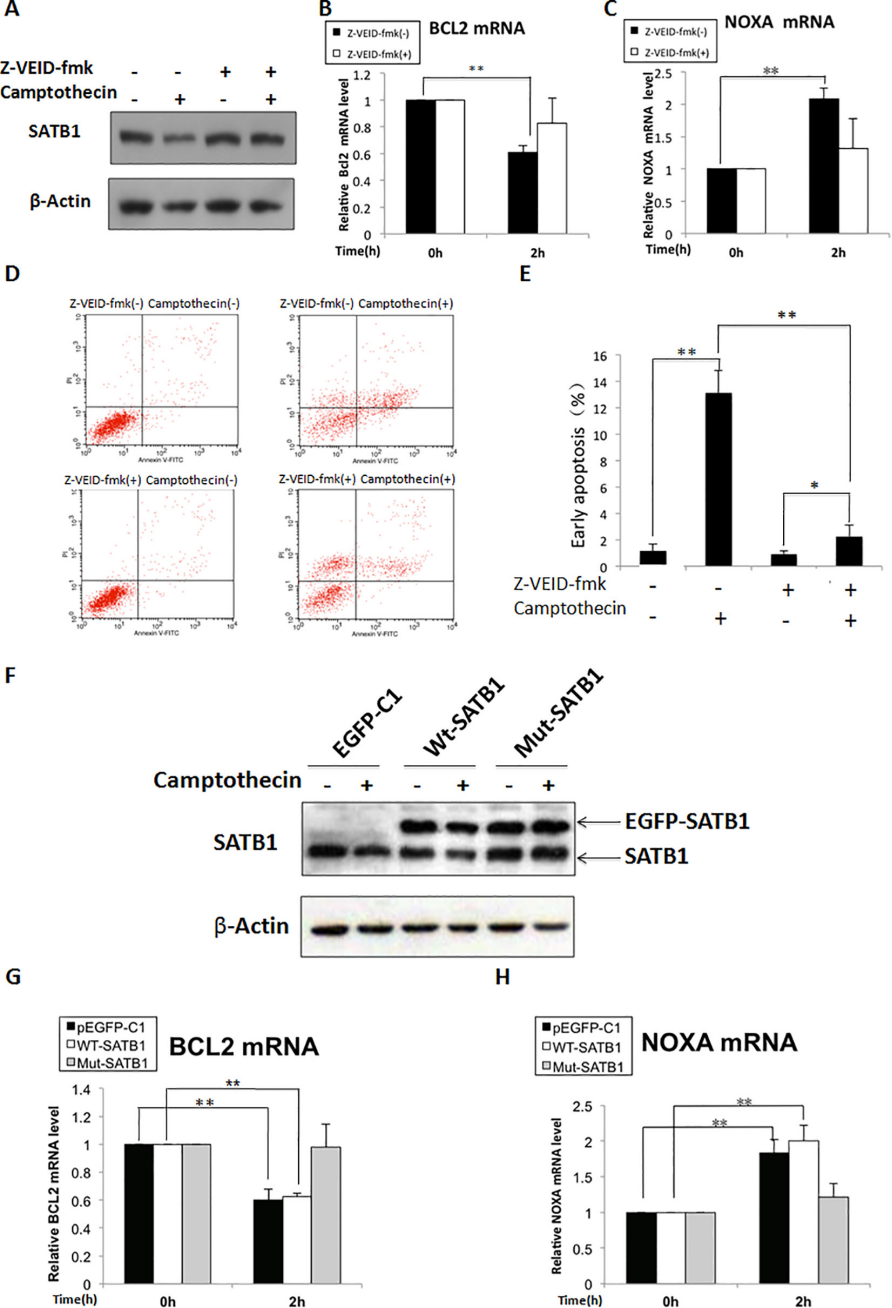
The specificity of SATB1 functions in co-regulating the expression of the BCL2 and NOXA genes in the cellular response to apoptosis. Cells were pre-incubated with 10μM Z-VEID-fmk, a specific inhibitor of caspase-6, for 30 min, before camptothecin treatment to inhibit SATB1 degradation. SATB1 levels were determined by Western blot (A). RT–PCR analysis revealed that inhibition of SATB1 degradation reversed the mRNA levels of both BCL2 and NOXA in camptothecin-treated cells (B & C), and significantly suppressed apoptosis (D & E). The specificity of SATB1 function was further confirmed by the expression of mutant SATB1 that was resistant to caspase-6 cleavage. Jurkat cells were transiently transfected with the pEGFP-C1 control vector、wild-type SATB1 expression vector (pEGFP-C1- SATB1), and mutant SATB1 expression vector, respectively, and treated with 5μM camptothecin for 2h to induce apoptosis 24 hours after transfection. SATB1 expression levels were determined by Western blot analysis, which showed that the expression level of mutant SATB1 was consistent in camptothecin-treated cells, and wild-type SATB1 was decreased (F). RT–PCR analysis showed that overexpression of mutant SATB1 completely restored BCL2 expression and inhibited the increase in NOXA expression, and overexpression of the wild-type SATB1 partly reversed mRNA levels of these two genes (G, H). The statistical differences were calculated using t-test. ‘**’ Represents *P*<0.01. The error bars represent standard deviation (*n* = 3).

SATB1 is the target of caspase-6 and is specifically cleaved by this enzyme during early apoptosis. In order to determine the specificity of the SATB1 function in the cooperative regulation of the BCL2 and NOXA genes during apoptosis, we examined the effects of inhibiting SATB1 degradation on BCL2 and NOXA transcriptional activity. Jurkat cells were pretreated for 30 min with 10 μM Z-VEID-fmk, a caspase-6 inhibitor, before the addition of camptothecin. Western blot analysis showed that pretreatment of cells with 10 μM Z-VEID-fmk completely inhibited camptothecin-induced cleavage of SATB1 (Fig 5). As expected, inhibition of SATB1 degradation reversed the mRNA levels of both BCL2 and NOXA in these cells (Fig 5), significantly suppressing apoptosis (Fig 5). The specificity of SATB1 function was further evaluated by an experiment using a mutant SATB1 expression vector that was resistant to caspase-6 cleavage. Jurkat cells were transfected with a wild-type SATB1 expression vector (EGFP-SATB1), a mutant SATB1 expression vector (EGFP-SATB1-D254A), or a pEGFP-C1 control vector before treatment with camptothecin. The transfection efficiency was examined by Western blot (Fig 5). As expected, overexpression of the mutant undegradable SATB1 almost completely reversed changes in expression levels of the BCL2 and NOXA mRNA in Jurkat cells treated with camptothecin, while overexpression of the wild-type SATB1 had only slight effects on the mRNA levels of these two genes under the same experimental conditions (Fig 5). These data clearly demonstrated that SATB1 plays a specific role in regulating the cooperative response of the BCL2 and NOXA genes to apoptotic stimulation.

## Discussion

Our previous studies demonstrate that SATB1 enhances BCL2 transcription by establishing chromatin loop architecture and recruiting cofactors to form an active transcriptional complex [25]. SATB1-mediated long-range chromosomal interaction between the mbr enhancer and BCL2 promoter is indispensable for high transcriptional activity of the BCL2 promoter[23] [24]. In the present study, we further investigated the function of SATB1-mediated higher-order chromatin structure in the cooperative regulation of the anti-apoptotic BCL2 gene and the pro-apoptotic NOXA gene. Our data showed that the mbr enhancer targeted the NOXA gene located 3.4Mb downstream of the BCL2 gene on chromosome 18.

The mbr enhancer, BCL2 promoter, and NOXA promoter were spatially tethered together through SATB1-mediated chromatin loop structures. The SATB1-triggered chromatin conversion regulated cooperative expression of the genes. The function of the chromatin conversion was supported by negatively coupled changes in mbr-BCL2 chromatin looping and mbr-NOXA chromatin looping that kept pace with the changes in the transcriptional activities of these two genes, induced by the altered SATB1 levels. As indicated by our q-ChIP, q-3C, and real-time PCR results, knocking down SATB1 expression with shRNA significantly reduced the SATB1 binding on the mbr enhancer and gene promoters, and switched the BCL2 chromatin loop to the NOXA chromatin loop. The decreased BCL2 chromatin looping and BCL2 expression were coupled with increased NOXA chromatin looping and NOXA expression. Our results provide the first evidence that the two Bcl2 family members with antagonistic functions share the same enhancer and were co-regulated through SATB1-induced higher-order chromatin conformation conversion.

SATB1 was not the only protein responsible for the re-organization of the BCL2 and NOXA chromatin loops, although it was critical for maintaining the BCL2 chromatin loop and the “mbr-BCL2-NOXA” chromatin structure/complex. It is still unclear why this looping not susceptible to SATB1 degradation and how the mbr and NOXA promoter were further tethered and maintained when the SATB1/SATB1 protein complex departed from the bases of the chromatin loops and the mbr-BCL2 chromatin loop was disassembled. New protein(s) must participate in further tethering the mbr-NOXA interaction and maintain the NOXA chromatin loop at higher levels after the departure of the SATB1/SATB1 complex. We propose that a potential candidate protein might be a transcription factor that could function as chromatin organizer by forming protein polymers and recruiting other transcription factors and cofactors to the binding sites. A spectrum of MAR-binding proteins, including Cux/CDP [27], SAF-A [28], Bright [29], SATB1 [30], and SATB2 [31] have been found to participate in the regulation of chromatin structure and gene transcription. It will be informative to investigate the roles of these MAR-binding proteins, especially the SATB1 homolog SATB2, in the co-regulation of the NOXA and BCL2 genes and other Bcl2 family members. CDP, a homeodomain protein of SATB1 that has a DNA-binding domain similar to SATB1 and competes with SATB1 to bind with specific DNA sequences [32], may be an especially promising candidate for the reorganization of the BCL2 and NOXA chromatin loops. Clarifying the functions of CDP and other possible candidate proteins in the chromatin conversion will provide insight into the rules governing apoptotic-stimulus-responsive dynamic changes in higher-order chromatin structures and their relationship to co-regulation of genes. It is also interesting that NOXA is looped with the mbr-enhancer in absence of apotosis. Although NOXA is a typical pro-apoptotic protein and plays an important role in regulation of apoptosis, NOXA expression can be detected under normal culture condition in the cell model we used, indicating the baseline NOXA expression is required for cell activity in absence of apoptosis. We speculate that the SATB1-mediated looping of NOXA with the mbr enhancer is required for keeping the baseline NOXA expression. Furthermore, the NOXA-mbr looping may provide a ready state which is helpful for the high expression of NOXA in cellular response to apoptosis stimulation.

The cooperative regulation of the BCL2 and NOXA genes by SATB1 at higher-order chromatin structure levels is significant in view of the profound association of SATB1 with cancer development and chemotherapy resistance [14,16]. In the present study, we carefully evaluated the biological significance of the SATB1-mediated chromatin loop conversion in apoptosis in Jurkat cells. SATB1 is known to be the target of caspase-6 [33]. We demonstrated that SATB1 cleavage at an early stage of apoptosis switched the BCL2 chromatin loop structure toward NOXA chromatin loop structure by modulating the association of SATB1 with the mbr and the two promoters. Consequently, BCL2 transcription was dramatically down regulated and NOXA transcription was up regulated.

It is important to point out that the role of SATB1 was specific, since transfection of cells with mutant SATB1 that were resistant to caspase-6 or treatment with caspase-6 inhibitor restored BCL-2 expression and reduced NOXA levels in Jurkat cells treated with camptothecin, and thus significantly suppressed apoptosis. Therefore, the SATB1 level profoundly influenced cell fate by co-regulating expression of the anti-apoptotic BCL2 gene and the pro-apoptotic NOXA gene during the cellular response to apoptosis. Recent studies have shown that many types of cancer cells aberrantly express SATB1 [9], which is positively correlated with poor prognosis and pathological properties [34,35]. The underlying mechanism is still unclear. Our data led us to infer that such aberrant SATB1 expression might endow cells with excess ability to resist apoptosis by maintaining the higher-order chromatin structure in favor of the expression of anti-apoptotic genes such as the BCL2 gene, thus suppressing pro-apoptotic genes, and consequently providing the cancer cells with selective advantages.

Genome-wide investigation of chromatin interaction and the interactome has revealed widespread stimulus-responsive promoter-promoter and promoter-enhancer interactions between co-regulated genes. This suggests the role of such a “multi-gene complex” in providing a topological framework for the transcription of co-regulated genes [36]. The intrinsic pathway of apoptosis is governed by the balance between opposing factions of the Bcl2 protein family that consists of pro-survival proteins and pro-apoptotic proteins. However, little is known about the function of higher-order chromatin structures in governing the balance of Bcl2 family members, although multiple transcriptional factors like CREB, ER alpha, and p53 [37–39] have been described to specifically regulate expression of this gene family and contribute to the control of the apoptosis pathway. The present study uncovered a novel mechanism with strong evidence that the BCL2 and NOXA genes were cooperatively regulated through SATB1-induced chromatin loop conversion, despite the antagonistic functions of these two genes. This finding suggests that different Bcl2 family members and other functionally related genes might be organized into one or more related frameworks by SATB1 or other transcription factors at higher-order chromatin structure levels, in order for their cooperative expression to regulate apoptosis. The mbr enhancer might be an important node of this framework. Further investigations combining 4C and high throughput techniques, such as ChIP-PET, to identify more SATB1-mediated mbr-promoter interactions and promoter-promoter interactions will provide new opportunities for clarifying how apoptosis is disrupted in cancer cells. In summary, our present study uncovered a novel mechanism of how the anti-apoptotic BCL2 and pro-apoptotic NOXA genes were cooperatively expressed by SATB1 at higher-order chromatin structure levels. Our work provides a valuable model for the investigation of SATB1-mediated long-range chromatin interactions in regulating the dynamic equilibrium of apoptosis-controlling genes with antagonistic functions.

## Supporting Information

S1 Fig The specificity of the PCR product generated by 3C technology designed to detect mbr and NOXA promoter interaction was validated by DNA sequencing. (TIFF)

S2 Fig The specificity of the PCR product generated by 3C technology designed to detect BCL2 and NOXA promoters interaction was validated by DNA sequencing. (TIFF)

S3 Fig 3C Analysis of the interaction between the mbr and the NOXA-1/NOXA-2 in vivo. The scheme of NOXA gene with the positions of restriction sites and positions of primers were indicated in A. The three SATB1 binding sites as been showed as N-SBS1、N-SBS2 and N-SBS3 (A). The 3C experiments were performed as described in the ‘Materials and Methods’ section. Physical interactions between mbr and NOXA-1/NOXA-2 were then determined by specific PCRs that detected hybrid fragments containing either mbr sequences or NOXA-1/NOXA-2 sequences. 3C data demonstrated that the mbr specifically interacted with NOXA-1 (B, upper panel), but not with NOXA-2 (B, middle panel). PCR products from HindIII digested cross-linked chromatin without ligation and non-crosslinked genomic DNA with or without ligation were used as negative controls. The bands shown in the bottom panels of B represented the PCR products from genomic DNA that was not cut by any restriction enzyme, which were used as the loading control. The bands shown in C are PCR products from BAC plasmids that showed the primers for 3C experiments are pretty good.
(TIFF)

S4 Fig The specificity of the PCR product generated by 3C technology designed to detect NOXA promoter downstream region and mbr interaction was validated by DNA sequencing. (TIFF)

S5 Fig The SATB1 expression level in the stimulation of camptothecin. (A) Western blot determined SATB1 expression level in Jurkat cells treated with camptothecin for three trials. (B) Gray analysis of SATB1 expression level in the stimulation of camptothecin showed SATB1 degraded to 70%. (C) SATB1 mRNA level decreased to 50% in the stimulation of camptothecin.
(TIFF)

S1 Table All the primers used in this article. (PDF)
