# Supplementary figures and images for: SATB1 Mediates Long-Range Chromatin Interactions: A Dual Regulator of Anti-Apoptotic BCL2 and Pro-Apoptotic NOXA Genes
Source: PLoS One. 2015 Sep 30;10(9):e0139170. doi: 10.1371/journal.pone.0139170 (PMC4589335; doi:10.1371/journal.pone.0139170)

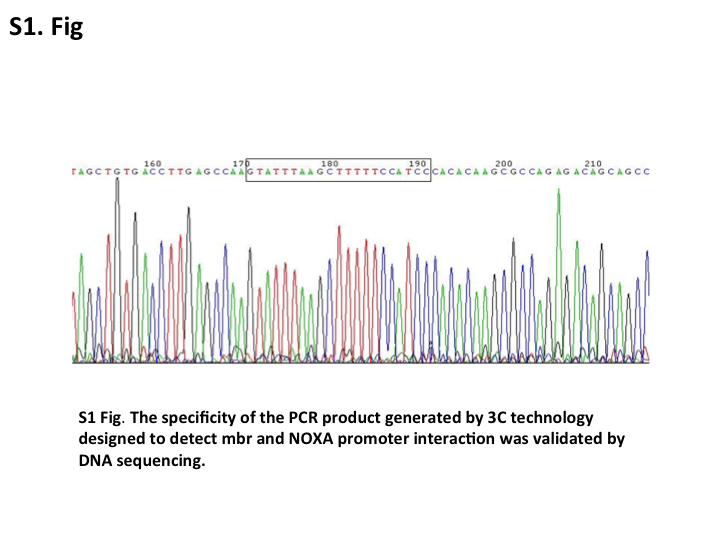

Supplement: S1 Fig — (TIFF) [file pone.0139170.s001.tiff]

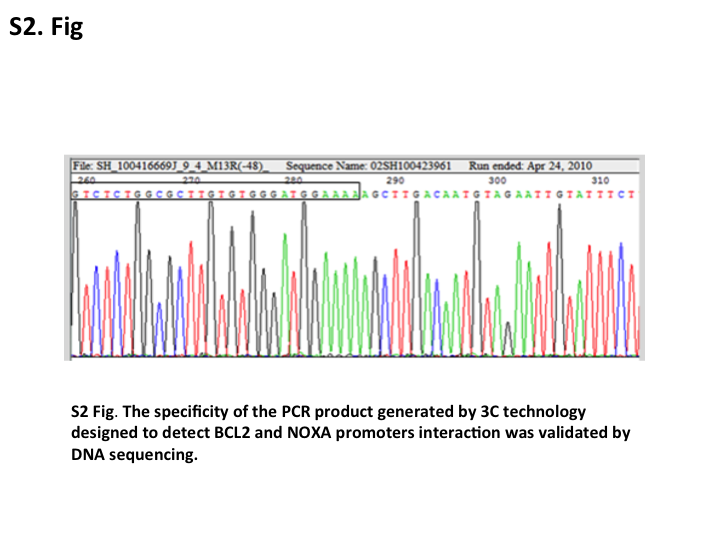

Supplement: S2 Fig — (TIFF) [file pone.0139170.s002.tiff]

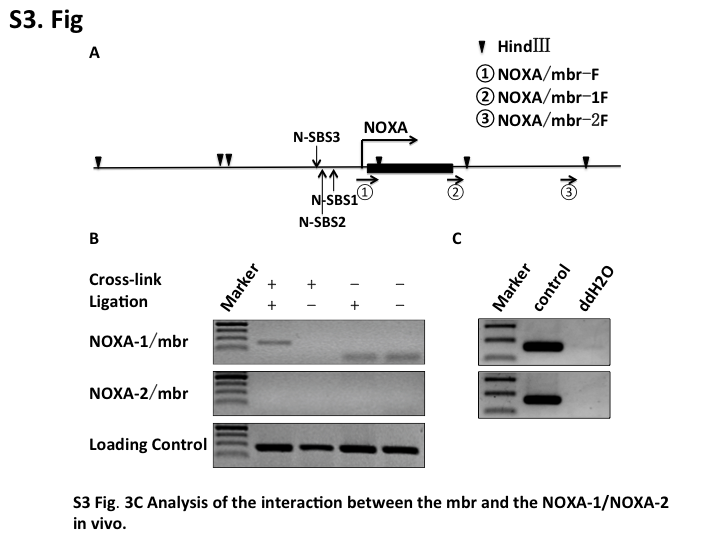

Supplement: S3 Fig — The scheme of NOXA gene with the positions of restriction sites and positions of primers were indicated in A. The three SATB1 binding sites as been showed as N-SBS1、N-SBS2 and N-SBS3 (A). The 3C experiments were performed as described in the ‘Materials and Methods’ section. Physical interactions between mbr and NOXA-1/NOXA-2 were then determined by specific PCRs that detected hybrid fragments containing either mbr sequences or NOXA-1/NOXA-2 sequences. 3C data demonstrated that the mbr specifically interacted with NOXA-1 (B, upper panel), but not with NOXA-2 (B, middle panel). PCR products from HindIII digested cross-linked chromatin without ligation and non-crosslinked genomic DNA with or without ligation were used as negative controls. The bands shown in the bottom panels of B represented the PCR products from genomic DNA that was not cut by any restriction enzyme, which were used as the loading control. The bands shown in C are PCR products from BAC plasmids that showed the primers for 3C experiments are pretty good. (TIFF) [file pone.0139170.s003.tiff]

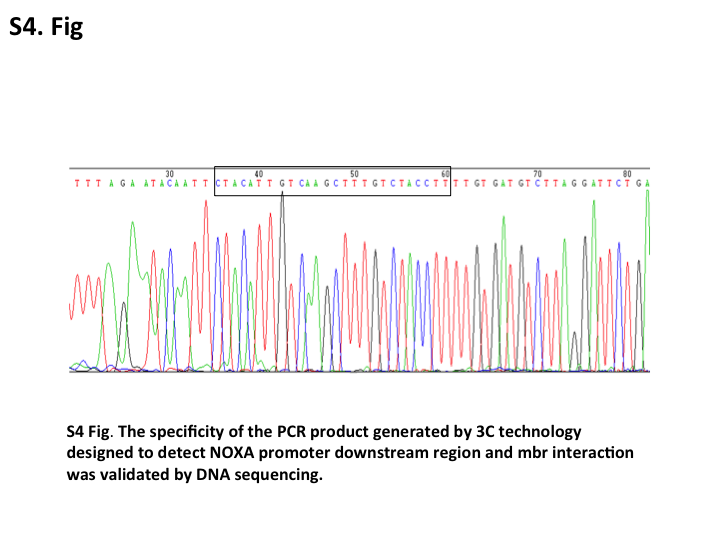

Supplement: S4 Fig — (TIFF) [file pone.0139170.s004.tiff]

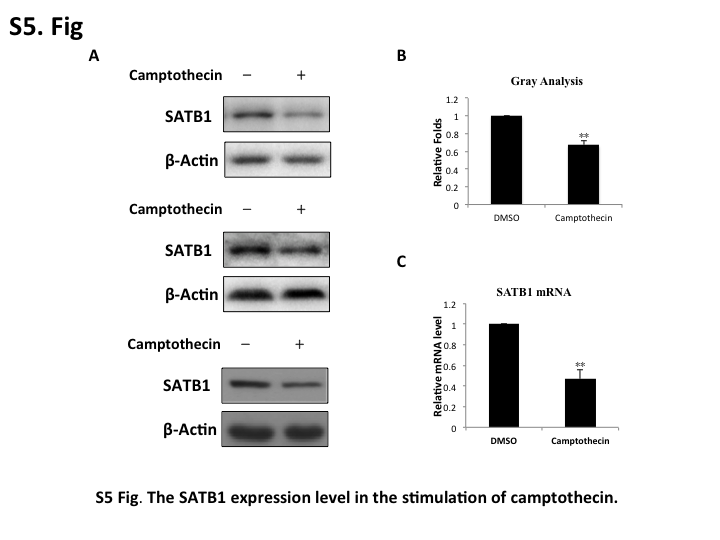

Supplement: S5 Fig — (A) Western blot determined SATB1 expression level in Jurkat cells treated with camptothecin for three trials. (B) Gray analysis of SATB1 expression level in the stimulation of camptothecin showed SATB1 degraded to 70%. (C) SATB1 mRNA level decreased to 50% in the stimulation of camptothecin. (TIFF) [file pone.0139170.s005.tiff]
