# Supplementary material for: SATB1 Mediates Long-Range Chromatin Interactions: A Dual Regulator of Anti-Apoptotic BCL2 and Pro-Apoptotic NOXA Genes
Source: PLoS One. 2015 Sep 30;10(9):e0139170. doi: 10.1371/journal.pone.0139170 (PMC4589335; doi:10.1371/journal.pone.0139170)
Supplement: S1 Table — (PDF) [file pone.0139170.s006.pdf]

Table S1. Primers used in the present study.

Primers		Sequence (5'-3')
ChIP	NOXA-SBS1-F	GTTGCCTAAGGTTTGTAGCCAG
	NOXA-SBS1-R	TCCAGGCTCATTTTGACTTACC
	NOXA-SBS2-F	CATTTTTGAACTTGTTTCACGA
	NOXA-SBS2-R	GGTATCATTTAATGTTGGGCTT
	NOXA-SBS3-F	ACAAACATGCTATACAGGAAAACAA
	NOXA-SBS3-R	AACCACAATGACAAAACAAAATAAG
	BCL2-SBS1-F	GGCATCTTGGCTATAATCAGTC
	BCL2-SBS1-R	GCCCAAAGACGAGTAGTCAA
	mbr-F	GCAGGATAGCAGCACAGGATT
	mbr-R	GGCCTCAGGGAACAGAATGAT
3C	BCL2/mbr-F	AATTCCATAGACAGGGGTCAAT
	BCL2/mbr-R	TTGCACTCGAGCCCTATTAAG
	AseI-PGK1-F	ATGTCCCTGGAGGTATCAATCT
	AseI-PGK1-R	GCAGCCACTACTCAGGAAGC
	NOXA/mbr-F	GATCTTATTCTGTGGTGTCTTTTG
	NOXA/mbr-R	TCTAAATCTAAACAGAGAACCCAGT
	NOXA/BCL2-F	TAACATTCCTTCTGTGCTGCTA
	NOXA/BCL2-R	TCTAAATCTAAACAGAGAACCCAGT
	NOXA-1/mbr-F	CGGACCAGAAAGTGCTATGC
	NOXA-1/mbr-R	GATCTTATTCTGTGGTGTCTTTTG
	NOXA-2/mbr-F	ACACCGCAGGGTATGTCTATCA
	NOXA-2/mbr-R	GATCTTATTCTGTGGTGTCTTTTG
	HindIII-PGK1-F	CTTCTCTTTCACCTCTACCCCT
	HindIII-PGK1-R	TTACAGTATAATACAAAACTCTCCAA
RT-PCR	BCL2-RTN-F	TCGCCCTGTGGATGACTGAG
	BCL2-RTN-R	CAGAGTCTTCAGAGACAGCCAGGA
	NOXA-RTN-F	GCAGAGCTGGAAGTCGAGTGT
	NOXA-RTN-R	CTCTTTTGAAGGAGTCCCCTCAT
	Actin-RTN-F	TCATGAAGTGTGACGTGGACAT
	Actin-RTN-R	CTCAGGAGGAGCAATGATCTTG
